# Factors Associated with Device, Internet and Videoconferencing Use Amongst Individuals with Moderate-to-Severe Traumatic Brain Injury

**DOI:** 10.3390/healthcare12141371

**Published:** 2024-07-09

**Authors:** Sarah L. Chuah, Diane L. Whiting, Thomas M. Gates, Grahame K. Simpson

**Affiliations:** 1 Brain Injury Rehabilitation Research Group, Ingham Institute for Applied Medical Research, Sydney, NSW 2170, Australia; sarah.chuah@health.nsw.gov.au (S.L.C.); diane.whiting@health.nsw.gov.au (D.L.W.); thomas.gates@health.nsw.gov.au (T.M.G.); 2 School of Psychology, University of Wollongong, Wollongong, NSW 2500, Australia; 3 School of Health Sciences, Faculty of Medicine and Health, University of Sydney, Sydney, NSW 2006, Australia; 4 John Walsh Centre for Rehabilitation Research, Northern Sydney Local Health District, Sydney, NSW 2065, Australia

**Keywords:** traumatic brain injury, videoconferencing, telehealth, telerehabilitation, internet, technology, computer anxiety, computer self-efficacy, technology fluency, technology skill, digital divide

## Abstract

Videoconferencing (VC) has the potential to improve access to quality healthcare for individuals with traumatic brain injury (TBI) who require intensive and ongoing rehabilitation post-injury. Gaps in information and communication technology (ICT) use, access, and skills, however, may undermine equitable participation in remotely delivered healthcare and rehabilitation. This cross-sectional study sought to identify which demographic, injury-related, and psychological factors are associated with gaps in digital inclusion amongst individuals with a TBI. Between March 2020 and December 2023, 186 adults with a moderate-to-severe TBI who were aged 18–65 years and were within five years post-injury completed a range of self-report measures. The results demonstrated that most individuals with a moderate-to-severe TBI reported high levels of technology skills and access and used the internet from multiple devices daily. While injury severity was unrelated to technology use, this finding may reflect an overestimation of technology use amongst individuals with the most severe injuries, who were excluded from the study. Several demographic and psychological factors were found to predict VC readiness and are presented within a model to guide clinicians considering client suitability for VC rehabilitation. The current findings indicate that the use of VC in clinical settings following a moderate-to-severe TBI is feasible and suggest that individuals with a TBI may benefit from the greater provision of remotely delivered healthcare than is currently offered.

## 1. Introduction

The use of internet videoconferencing (VC) in healthcare has increased rapidly following the global SARS-CoV-2 pandemic [1]. Previously, VC-delivered health services were prioritized for rural and remote populations; however, they are now widely offered as an alternative or adjunct to the assessment and treatment of a range of acute and chronic conditions [2,3], with levels of patient satisfaction and clinical outcomes equivalent to standard care [4,5,6,7,8,9]. While VC has the potential to bridge the healthcare divide for vulnerable populations, gaps in information and communication technology (ICT) engagement may undermine equitable participation in remotely delivered healthcare.

Traumatic brain injury (TBI), resulting from an external force applied to the brain, commonly due to motor vehicle accidents, assaults, falls, and sporting injuries, is a leading cause of disability worldwide [10,11,12]. The injury severity ranges across mild, moderate, and severe levels and can be measured by the duration of post-traumatic amnesia (PTA), a period where brain function is altered, featuring disorientation and disruption to memory function [13]. Moderate-to-severe injuries, the focus of this report, can result in an array of physical, cognitive, emotional, and behavioral sequelae requiring long-term rehabilitation [14]. Individuals with a traumatic brain injury (TBI) have the potential to benefit greatly from the clinical use of VC, as rehabilitation following TBI requires specialized, integrated, interdisciplinary care over an extended post-injury period [15]. While telehealth avenues such as VC are gaining recognition as a feasible, cost-effective means of accessing healthcare following a brain injury [16,17,18,19,20], the provision of VC appointments has been limited within brain injury rehabilitation [21,22], indicating a potential gap in the clinical application of VC with this population.

The “digital divide” literature that has burgeoned globally over the past 20 years [23] examines gaps in technological inclusion and provides a useful lens through which to examine VC use amongst individuals with a TBI. At the most basic level, a digital divide may result from gaps in access to ICT infrastructure (level one divide); however, once access to technology becomes omnipresent, gaps in technology use (e.g., experience and skill; level two divide), arising from individual or systemic constraints, represent a more nuanced form of digital exclusion and can explain subsequent gaps in how individuals derive benefits from technology in their everyday lives (e.g., use for productive vs. leisure purposes; level three divide). Existing research indicates that individuals with a TBI may experience a digital divide on multiple levels. For instance, individuals with a TBI are less likely to use the internet, have greater difficulty completing tasks online, and are less likely to receive clinical services remotely via VC than the general population [22,24,25].

Several demographic factors, including age, education, and occupation, appear to be important indicators of digital inclusion in the general population. Higher education attainment has been associated with greater internet use and technology skills, while, in relation to employment status, those employed, unemployed, or undertaking study have demonstrated higher levels of technology skill relative to homemakers and retirees [26,27,28]. Other factors, including the female sex, a lower income, a minority race/ethnicity, and a non-urban geographical area, have also been associated with the digital divide [28,29,30,31].

Within the TBI literature, limited studies have explored the factors associated with technology use; however, the research lacks clarity. With regard to age, older individuals who have sustained a moderate-to-severe TBI and stroke appear to be less likely to use the internet, mobile devices such as smart phones and tablets, and smartphone applications (“apps”), relative to younger individuals [28,32,33,34]. While service providers have cited cognitive impairment and technological capacity as major barriers to VC use with brain injury clients [21,22,35], other research indicates that lower levels of executive functioning are associated with the greater use of mobile devices in order to provide a scaffold for cognitive impairments [34]. Importantly, no studies to date have explored the relationship between TBI severity and technology use or skills.

Personal attitudes and psychological responses towards technology affect engagement with technology [36] and are likely to be important variables to consider when determining readiness for VC in individuals with a TBI. For example, low computer self-efficacy, a measure of one’s confidence in using computers, has been associated with higher computer anxiety [37,38] and lower technology use in non-injury samples [36,39]. Higher levels of computer anxiety may lead to avoidance of technology and result in fewer opportunities to acquire technology skills or fluency, increasing participation restrictions and contributing to poorer outcomes for individuals [40,41].

With the increased availability of VC telehealth appointments since the global SARS-CoV-2 pandemic, a better understanding around client readiness for VC rehabilitation after a TBI is warranted. While a substantial body of research has explored the factors associated with the digital divide in the general population over the past two decades, limited research has explored the factors associated with the digital divide in a TBI population. There is a need to better understand which factors contribute to an individual’s readiness to engage in TBI rehabilitation via VC, considering possible cognitive impairments and elevated psychological distress when compared to the general population.

This study aims to improve our understanding of individuals’ readiness to participate in VC telehealth appointments post-TBI by (i) determining post-injury levels of device use, internet access, and VC experience among people with a moderate-to-severe TBI; (ii) identifying post-injury levels of technology skill (fluency) and technology-related psychological factors including computer anxiety and computer self-efficacy in this population; (iii) identifying the demographic, injury-related, and psychological factors associated with technology use, access, and skills post-injury; and (iv) developing a model of VC readiness outlining the interactions between these factors, which will allow clinicians to identify potential barriers for an individual considering engaging in rehabilitation via VC.

## 2. Materials and Methods

### 2.1. Sample and Setting

Participants were included in the present cross-sectional study if they were aged 18–65 years and had sustained a moderate-to-severe TBI (as indicated by a medically documented PTA period of 7 days or more), after the age of 18 and within the past 5 years. Individuals were excluded from the study if they were deemed by their medical team as lacking the cognitive capacity to respond to self-report measures (e.g., they were in a current or chronic amnesic state) or not having adequate English or communication skills to respond to the survey questions. The study was granted ethics approval for a period of five years, through the South Western Sydney Local Health District Human Research Ethics Committee (reference number: 2019/ETH13152),and was conducted between March 2020 and December 2023.

The study was undertaken within the New South Wales (NSW) Health Brain Injury Rehabilitation Program (BIRP) in Australia [42]. This public program is the lead provider of specialist brain injury rehabilitation services within NSW, comprising a network of 15 inpatient, transitional, and community-based rehabilitation services for children and adults of working age. All patients who are identified as having sustained a greater than moderate TBI in NSW initially enter the BIRP for specialist care and may later go on to receive private services once discharged from inpatient services. The current study was conducted among the adult community Brain Injury Rehabilitation Units (BIRUs) in metropolitan (Liverpool, Westmead, Illawarra, Hunter) and regional (Mid-Western, South West, Southern Area, Mid-North Coast) NSW.

### 2.2. Measures

Participants completed a battery of self-report measures collecting routine demographic and injury-related information; a study-specific technology use, access, and experience questionnaire; and four validated measures exploring technology-related variables (i.e., technology fluency, computer anxiety, and computer self-efficacy) and psychological distress.

***Technology Use, Access, and Experience Questionnaire***

Participants were first asked to report on their current (i.e., post-TBI) use of digital devices including smartphones, standard mobile devices, tablets, and personal computer (PC) devices. For each device used, participants were asked to indicate whether they accessed the internet from the device and if the device had functionality suitable for VC calls (i.e., if the device had a working camera, microphone, and speaker). Participants were then asked whether they had any pre- and post-injury experience using various popular VC platforms and were invited to report on any other VC platforms used (pre- and post-injury). Approximately halfway through the study, the research team expanded the focus to gather additional data on internet use frequency and experience using VC for telehealth purposes, resulting in a subset of participants (*n* = 91) indicating their internet use frequency (ranging from multiple times per day to once per month or less), the frequency of VC use for telehealth (ranging from weekly to rarely), and the health professionals seen via VC.

***Technology-Related Psychological Variables***

The below measures were used to assess a range of current (i.e., post-injury) technology-related psychological variables.

*Computer-Email-Web Fluency Scale* (CEWFS) [43]

A 21-item self-report scale that self-assesses an individual’s perceived technology fluency (skills) around a variety of computer-, email-, and web-related tasks, on a five-point Likert scale (1 = not at all, 5 = very well). The CEWFS encompasses four technology constructs: computer fluency, e-mail fluency, web navigation, and web editing. While each of these scores was calculated for descriptive analyses, we focused on the total score as a measure of the overall technology skill to indicate VC readiness. Total scores range from 21 to 110, where higher scores indicate higher levels of technology skill. Some modifications to the wording of the measure were made to make it more current (i.e., “I can use search engines such as Yahoo or Alta Vista” was changed to “I can use search engines such as Yahoo or Google”, “I can save text contents/images off Web pages to a disk” was changed to “I can save text contents/images off Web pages to a device”, and “I can use a browser such as Netscape or Explorer to navigate the World Wide Web” was changed to “I can use a browser such as Explorer or Chrome to navigate the internet”. Perceived technology fluency has been shown to be significantly related to actual fluency (*r* = 0.23), as measured by applied computer skills, and is significantly associated with computer anxiety (*r* = −0.42) [44]. The CEWFS has demonstrated good internal consistency as a measure of technology fluency in a student population (α = 0.89) [43].

*Short Computer Anxiety Scale* (SCAS; [45])

A six-item scale investigating computer anxiety, featuring two items about comfort and four items associated with inadequacy in using computers. The scale uses a six-point Likert-scale (1 = strongly disagree, 6 = strongly agree), with total scores ranging between 6 and 36, where higher scores indicate greater computer anxiety. The scale has demonstrated moderate internal consistency in student populations, with a Cronbach’s alpha of 0.78 [45].

*Modified Computer Self-Efficacy Scale* (mCSES; [46])

A modified version of the CSES [47] assessing confidence in using novel computer devices amongst older adults and individuals with a disability. The mCSES is a 10-item scale assessing confidence under a range of conditions using a 10-point Likert scale (where 1 = not at all confident, 10 = completely confident). It provides a total score between 10 and 100, where higher scores indicate greater computer self-efficacy. The scale has demonstrated high internal consistency with a standardized alpha coefficient of 0.94 [46]. Minor modification to the wording of the mCSES was applied to make it more relevant to computer devices (e.g., the word “product” was changed to “device”).

***Psychological Distress***
*Depression Anxiety and Stress Scale-21* (DASS-21) [48]

A 21-item questionnaire that assesses the constructs of depression, anxiety, and stress over a seven-day period on a four-point Likert scale (0 = never, 3 = almost always). The DASS-21 is commonly used in clinical practice within Australia and provides a total score ranging from 0 to 63 as a broad measure of psychological distress, with higher scores indicating greater levels of psychological distress. The original factor structure of the DASS-21 has been replicated in samples with a moderate-to-severe TBI, with high internal consistency (α = 0.95) [49].

### 2.3. Recruitment and Data Collection

The active client database at each BIRU was screened by author SC or local BIRU staff to identify prospective participants, who were then reviewed by the assigned case manager and/or rehabilitation specialist to confirm their eligibility for the study. Local BIRU staff, known to the participants, made initial contact with prospective participants to obtain permission for the research staff to recruit to the study. Author SC then supplied the study information to participants via email, post, or telephone and participants provided informed consent verbally prior to completing the survey questions.

All participants completed the study measures via telephone, with the exception of one who completed the measures face-to-face with author SC at their home and another who received paper copies of the measures via post and mailed back completed forms in a pre-paid, self-addressed envelope. On average, the entire battery of measures took 30 min to complete.

### 2.4. Statistical Analysis

Statistical analysis was predominantly conducted using JMP v15 [50], with AMOS v26 [51] used to perform structural equation modeling (SEM). Descriptive statistics were generated to characterize the sample according to demographic, injury-related, and psychological factors, as well as their technology use, access, and skill (objectives (i) and (ii)). As most of these variables were non-normally distributed, non-parametric tests were used to assess which demographic, injury, and psychological factors were associated with device use, internet access, VC experience, and technology fluency (objective iii). For continuous data, this included Spearman’s ρ bivariate and point-biserial correlations or Kruskal–Wallis tests followed up post hoc with Bonferroni-corrected Mann–Whitney U tests to control the Type I error rate. Associations between count/categorical data were analyzed using χ^2^ tests. Effect sizes for correlations were interpreted according to Cohen’s convention: 0.1 = small, 0.3 = medium, 0.5 = large [52].

To address objective (iv), SEM was used to develop a Model of VC Readiness that accounted for the interactions between factors contributing to technology fluency and VC use post-injury. In the initial step, variables with univariate associations exceeding ρ = 0.3 were entered into the model. Additional pathways and variables were considered in subsequent steps if there was a strong theoretical basis for inclusion and they were found to improve the model fit based on the inspection of modification indices. The fit of the model was tested against common criteria of fit and parsimony, including absolute fit indices, namely χ^2^ (overall model fit, ns) and the root mean square error (RMSEA) with 90% CIs (<0.08 good) and the standardized root mean square residual (SRMR; <0.05 good), and incremental fit indices, namely the comparative fit index (CFI; >0.95 good), normed fit index (NFI; >0.95 good), and Tucker–Lewis index (TLI; >0.95 good) [53,54].

## 3. Results

### 3.1. Sample Characteristics

In total, 253 individuals were contacted, of whom 186 agreed to participate in the study (74% response rate). The demographic and injury characteristics of the sample are presented in Table 1. Age was bimodally distributed, with a younger peak around ages 20–30 and an older peak around ages 50–60. The majority (*n* = 159, 85%) of participants were born in Australia, with 11 (6%) reporting Indigenous status. One hundred and twelve (60%) participants resided in metropolitan NSW and the remaining 74 (40%) lived in regional areas.

Regarding the injury severity, 121 (65%) participants sustained a very severe TBI (defined as a PTA of 7–28 days), while the remaining 65 (35%) sustained an extremely severe TBI (>28 days PTA). Over half of the participants sustained their injuries in a motor vehicle or pushbike accident (*n* = 97, 52%), approximately one third (*n* = 69, 37%) from a fall, and the remaining 20 (11%) from other circumstances. Approximately one third (*n* = 69, 37%) were receiving financial compensation through a government-funded insurance scheme; the rest were not (*n* = 117, 63%). At the time of the study, 66 (35%) participants had successfully returned to work (*n* = 19 as professionals/managers, *n* = 47 in other employment categories), 76 (41%) were still actively involved in rehabilitation, and 44 (24%) were in supported employment or not working.

The median DASS-21 total score was 15 (IQR = 23; range= 0–56), with just over half of the sample (*n* = 97; 52%) reporting clinical levels of psychological distress (DASS-21 total score > 13). Of these, 66 participants (68%) were not currently receiving psychological support.

### 3.2. Device Use and Internet Access

Nearly all participants reported having access to the internet through a smartphone (*n* = 185/186, >99%), with the majority (*n* = 129, 69%) reporting having access to the internet from two or more devices. More participants reported that they used a desktop or laptop PC (*n* = 109, 59%) than a tablet device (*n* = 73, 39%) to access the internet. Of those asked the additional questions about the frequency of their internet use (*n* = 91), the majority (*n* = 86, 95%) reported regular internet use, with most (*n* = 80, 88%) indicating that they used the internet at least once per day and the remainder indicating that they used the internet at least once per week (*n* = 3, 3%) or less than weekly (*n* = 3, 3%). Almost two thirds (*n* = 118, 63%) of the participants reported having access to a device (in addition to their mobile phone) that was suitable for VC (i.e., had a functioning microphone, speaker, and camera).

### 3.3. VC Experience

Videoconferencing use was common across the sample, with 138 out of 186 participants (74%) reporting the use of at least one VC platform prior to their injury and a slightly higher proportion (*n* = 147, 79%) reporting that they had used VC since sustaining their injury.

Similar numbers of participants reported using one (*n* = 29, 16%), two (*n* = 35, 19%), three (*n* = 36, 19%), four (*n* = 24, 13%), or five plus (*n* = 23, 13%) different VC platforms post-injury. The most frequently used VC platform was FaceTime (*n* = 100, 54%), followed by Zoom (*n* = 81, 44%), Facebook Messenger (*n* = 63, 38%), Skype (*n* = 39, 21%), WhatsApp (*n* = 32, 17%), Microsoft Teams (*n* = 24, 13%), and SnapChat (*n* = 21, 11%).

Of those asked about their experiences with using VC in a healthcare context (*n* = 90), around half (*n* = 51, 57%) indicated that they had previously used VC for a telehealth appointment. Medical specialists (including rehabilitation physicians, psychiatrists, and neurologists) were the health professionals that most participants had seen for VC telehealth appointments (*n* = 26/85; 31%), followed by psychologists (*n* = 21; 25%) and speech pathologists (*n* = 12; 14%).

### 3.4. Technology Fluency, Computer Anxiety, and Computer Self-Efficacy

Overall, technology fluency was very high across the sample, with the computer and email skill subscale scores reaching the highest levels (Table 2). In contrast, participants reported moderate levels of computer anxiety, with median scores falling just below the midpoint of the measure. Computer self-efficacy was relatively high, with the median score falling well above the midpoint.

### 3.5. Factors Associated with Technology Variables

Univariate associations between the demographic factors and technology-related variables are displayed in Table 3 and Table 4. Regarding the demographic factors, males (66%) were less likely than females (88%) to access the internet from two or more devices (χ^2^ = 4.53; *p* = 0.03). They also reported using fewer VC platforms in total following their injury. Age was negatively associated with post-injury VC use, the number of VC platforms used, technology fluency, and computer self-efficacy, while being positively associated with computer anxiety. These correlations were all small to medium in size. Participants living with another person were more likely to have used VC post-injury than those who lived alone (corresponding to a small effect size), but the living arrangement was otherwise unrelated to technology access, use, and skills. Geographical remoteness was not associated with any technology variables (all *p* > 0.36).

Education was positively associated with all technology-related variables, except for computer anxiety, with which it was negatively correlated. These relationships reflected medium to large effect sizes. In relation to employment status, those working as professionals/managers at the time of injury reported superior technology access, use, and skills compared to those who were working in other occupational categories and those who were not working. For computer self-efficacy, the same trend emerged amongst the employment status categories but was only marginally significant (*p* = 0.054). These differences are likely to have been mediated by education, which was higher in professionals/managers compared to the other two pre-injury employment categories (H/χ^2^ = 39.38, *p* < 0.0001).

Psychological distress was negatively associated with the number of devices used to access the internet, computer self-efficacy, and technology fluency and positively associated with computer anxiety. These associations reached small to medium effect sizes.

Interestingly, the injury severity (i.e., PTA duration) was not associated with any technology-related variables (all *p* > 0.23).

All technology-related variables were strongly intercorrelated, with the strength of the effect sizes ranging from medium to very large (Table 5). The associations were all in the positive direction, except those involving computer anxiety, which was negatively related to the other variables.

### 3.6. A Model of VC Readiness

Missing data on the SCAS for item 5 (*n* = 5) were replaced using the scale mean. The replaced missing data gave the SEM a final sample size of *n* = 184. The following variables met the condition of having a correlation of at least ρ = 0.3 with another variable and were entered into the VC readiness model at the initial step: education, psychological distress, number of additional devices used to access the internet, post-injury VC use, computer anxiety, computer self-efficacy, and technology fluency. Sex and age were not included in the initial iteration of the model, but, as they were found to be significantly associated with at least one technology use or skill variable, they were considered in subsequent iterations if their inclusion further improved the overall model fit. The number of VC platforms used was not considered for inclusion due to its highly contingent relationship with post-injury VC use.

The examination of the variable distributions revealed a significant departure from multivariate normality (z_CR_ = 2.74, *p* = 0.003). To address this assumption, the model was re-run after linear transformations were applied to highly skewed variables (i.e., DASS-21, CEWFS, mCSES, and SCAS) to normalize their underlying distributions. However, substituting the transformed variables into the model did not improve its fit of the dataset. Therefore, the following section outlines the results of the model based on untransformed variables.

**Figure 1:**
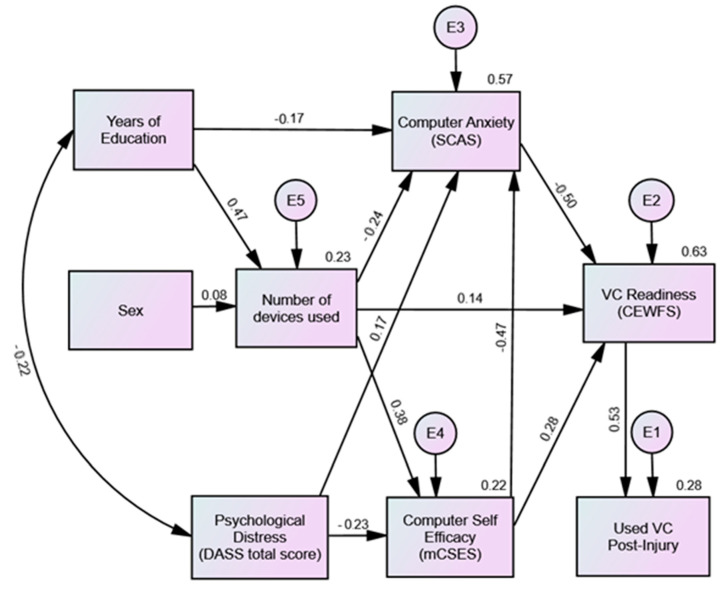
Final VC readiness model.

The final model is presented in Figure 1. It accounted for 63% of the variance in technology fluency (VC readiness) and 28% of the variance in post-injury VC use. All measures of the model fit fell within the recommended guidelines (Table 6). The model indicated that post-injury VC use was directly predicted by higher technology fluency (VC readiness), which was in turn indirectly predicted by higher levels of education, lower psychological distress, and the female sex, with the relationship mediated by computer anxiety, the number of additional devices used, and computer self-efficacy. Computer anxiety was a key feature of the model, with all other explanatory factors contributing to the scores on this measure.

All standardized path coefficients in the model were statistically significant (*p* < 0.005), aside from the pathway from sex to the number of additional devices used (*p* = 0.23). However, the removal of sex from the model led to a slightly poorer overall model fit (χ^2^(9) = 18.766, *p* = 0.027; RMSEA = 0.077 (0.019–0.123); SRMR = 0.049, NFI = 0.965, TLI = 0.955, CFI = 0.981).

## 4. Discussion

### 4.1. Clinical Implications

This study sought to determine the levels of technology use, access, and skills in a TBI sample and clarify which demographic, injury-related, and psychological factors are associated with the digital divide in this population. The high rates of VC use reported for personal and work purposes, together with the high levels of technology fluency and computer self-efficacy reported, suggest that individuals with a TBI may be underserved in telehealth applications of VC and could benefit more from the provision of remotely delivered healthcare than is currently available. Existing research indicates that individuals who access the internet from multiple devices demonstrate greater diversity in their internet use and are more likely to benefit from using the internet compared to those who only access the internet from a phone [55,56]. Consistent with these findings reported for the general population, the current study demonstrates how the number of devices used by individuals with a moderate-to-severe TBI affects computer self-efficacy and computer anxiety and impacts overall VC readiness.

Despite clinician-reported concerns about the cognitive effects of TBIs on clients’ suitability for eHealth and mHealth services such as VC-enabled telehealth [21,22,35], the present study found no relationship between the injury severity and internet, device, and VC use post-injury. Instead, health professionals may consider other demographic and personal factors in their assessment of a client’s readiness or suitability for VC telehealth services, including the number of devices that they use post-injury, their education, and their level of psychological distress.

### 4.2. Limitations

The study had limitations in both its design and delivery, along with changes in environmental factors during data collection. The design was cross-sectional and could not provide an indication of the development and learning in technology use that may occur over time post-TBI. In terms of delivery, the finding that technology use was unrelated to the injury severity may reflect an overestimate among people with the most severe injuries (e.g., those experiencing chronic post-traumatic amnesia), as those who were identified by the treating staff as not having the cognitive and/or language abilities to complete self-report measures were excluded from the study. Moreover, the study did not employ an objective measure of computer skills, and the TBI clients’ self-reports may also be subject to bias (e.g., where poor insight may result in the overestimation of their actual skills). In relation to environmental factors, the period covered was quite extensive (i.e., three years, between March 2020 and December 2023) and included a catastrophic change to living conditions brought about by the global SARS-CoV-2 pandemic. The pandemic led to the greater uptake of VC technology, and, once again, due to the cross-sectional nature of the study, some of the earlier results may underestimate the current post-pandemic technology use.

### 4.3. Future Research

The current study lays the foundation for some useful avenues of future research. We did not ask about the supported use of technology, and a future study could investigate whether people with TBIs who have family/carer support may be less impacted by the digital divide than those living alone. The study was not able to investigate other perceived barriers or additional psychological or sensory factors likely to impact technology use in this population (e.g., motivation, vision, and motor impairments), which may impact technology use (e.g., the more frequent use of memory apps has been associated with fewer motor symptoms and visual impairments following ABI [33,34]), and therefore further work examining these barriers is warranted.

Finally, it would also be useful to examine the types of internet use amongst the TBI population in greater detail. As the types of internet use vary in their level of productivity and benefit for individuals (e.g., for information, news, personal development, social interaction, leisure, commercial transaction, and gaming purposes), it would be useful to explore the level 3 digital divide in a TBI population to better understand where gaps in participation and inclusion may exist [57].

### 4.4. Key Takeaways

The results demonstrate that most individuals with a moderate-to-severe TBI report high levels of technology skill and access and use the internet from multiple devices daily. This high level of engagement with technology is consistent with the previously reported broad range of functions that technology serves for individuals with a TBI [32] and the general trend of personal devices becoming increasingly affordable, accessible, and central to everyday living in developed nations [23]. The reduced rates of VC for telehealth relative to personal and professional use identified in our sample indicate that there is greater potential to utilize VC in TBI rehabilitation and are consistent with the apparent service gaps in the delivery of VC telehealth appointments to individuals with a TBI [21,22]. It is worth noting, however, that the rates of VC for telehealth in the current study are higher than those reported in previous studies, a finding that is likely attributed to the broad pivot to online health services in response to the global SARS-CoV-2 pandemic.

In line with the broader digital divide literature, a range of demographic factors, including age, sex, education, and employment, appear to be similarly associated with technology access and use in the TBI population [23]. While geographical remoteness has previously been associated with various forms of digital divide in Australia and elsewhere [29,58], the current study’s finding that geographical remoteness was unrelated to technology access, use, or skills may be an indicator that access gaps in digital inclusion overall are reducing [23].

To our knowledge, the current study was the first to examine the role of brain-injury-specific factors in technology variables and found that the injury severity was unrelated to people’s use and access to technology. This is important, as it suggests that technology skills may be a form of preserved pre-injury knowledge, which could be an important resource for a broad range of people with a moderate-to-severe TBI post-injury. The study has also provided a comprehensive Model of VC Readiness for individuals with a moderate-to-severe TBI, accounting for 63% of the variance in VC readiness. The model highlights the relative influence that education, psychological distress, sex, computer anxiety, the number of devices used, and computer self-efficacy have on overall technology skill and VC use.

## 5. Conclusions

The results of this study indicate that the use of VC for telehealth and rehabilitation services for adults following a moderate-to-severe TBI is both feasible and likely underutilized, given the high levels of technology and VC use reported in this cohort. Despite clinician perceptions that the cognitive impacts associated with a moderate-to-severe TBI render clients less suitable for VC services, the current study found that the injury severity was not associated with technology skill and use post-injury, and it highlights the importance of considering a number of demographic and personal factors when clinicians are determining their client’s suitability for VC services, including education, psychological distress, sex, and the number of devices used post-injury.

## Figures and Tables

**Table 1:** Demographic and injury characteristics for participants (*n* = 186).

Variables		
Sex (male) (*n*, %)	153 (82)	
Age (Med, IQR, range)	42 (29)	18–65
Years of education (M, SD, range)	12.3 (2.1)	7–19
Time since injury (months) (Med, IQR, range)	16.5 (27)	1–60
PTA (days) (Med, IQR, range)	21 (23)	7–183
Pre-injury employment (*n*, %) Working—professional/manager Working—other vocational category Not working ^1^	40 (22) 118 (63) 28 (15)	
Current living arrangement (*n*, %) Living with others ^2^ Living alone	154 (83) 32 (17)	

Note. ^1^ Students (*n* = 4), volunteers (*n* = 1), and homemakers (*n* = 2) included in this category alongside avocational/retired (*n* = 21). ^2^ Includes living with spouse (*n* = 78), parents (*n* = 48), other family (*n* = 14), friends (*n* = 7), attendant care/nursing staff (*n* = 2), or other (*n* = 5).

**Table 2:** Technology measures ^1^.

Measures	Med (IQR)
Computer Anxiety (SCAS)	14.5 (11)
Modified Computer Self-Efficacy (mCSES)	73 (24)
Technology Fluency (CEWFS Total Score) Computer Skills Email Skills Web Navigation Web Editing	95 (23) 29 (8) 30 (3) 22 (6) 15 (9)

Note. ^1^ *n* = 180 for SCAS; *n* = 184 for mCSES and CEWFS.

**Table 3:** Spearman’s ρ bivariate and point-biserial correlations between demographic and technology use, access, and skill variables.

	No. of Devices Used	Post-Injury VC Use	No. of VC Platforms Used	Computer Anxiety (*n* = 180)	Computer Self-Efficacy (*n* = 184)	Technology Fluency (*n* = 184)
Sex (Female = 0, Male = 1)	−0.12	−0.14	−0.18 *	<−0.01	0.02	−0.07
Age	−0.02	−0.22 **	−0.24 **	0.16 *	−0.25 ***	−0.25 ***
Education	0.49 ****	0.32 ****	0.38 ****	−0.48 ****	0.31 ****	0.50 ****
Living Arrangement (Alone = 0, With Others = 1)	0.14	0.17 *	0.09	−0.10	0.10	0.12
Psychological Distress	−0.18 *	−0.13	−0.12	0.42 ****	−0.35 ****	−0.29 ****

Note. * *p* < 0.05 ** *p* < 0.01 *** *p* < 0.001 **** *p* < 0.0001. N = 186 unless otherwise specified.

**Table 4:** Associations between pre-injury employment status and technology variables.

	Pre-Injury Employment Status					
	Working— Professional/ Manager (*n* = 40)	Working—Other Occupational Category (*n* = 118)	Not Working (*n* = 28)	H/χ^2^	*p*	Post Hoc ^1^
No. of Devices Used (Med, IQR)	2 (1)	1 (1)	1 (1)	19.49	<0.0001	P/M > OW&NW
Post-Injury VC Use (*n*, %)	39 (98)	91 (77)	17 (61)	14.16	0.0008	P/M > OW > NW
No. of VC Platforms Used (Med, IQR)	3 (3)	2 (2)	1.5 (3)	15.40	0.0005	P/M > OW&NW
Computer Anxiety (*n* = 180) (Med, IQR)	10 (8)	16 (12)	14.5 (8)	11.46	0.003	P/M < OW
Computer Self-Efficacy (*n* = 184) (Med, IQR)	79 (21)	70.5 (31)	76.5 (27)	5.84	0.054	
Technology Fluency (*n* = 184) (Med, IQR)	99 (7)	93 (26)	90.5 (21)	10.41	0.006	P/M > OW

Note. *n* = 186 unless otherwise specified. ^1^ Kruskal–Wallis tests used to assess overall effect. Post hoc testing of significant overall effects was conducted using pairwise Mann–Whitney U tests with Bonferroni-corrected critical α = 0.0167.

**Table 5:** Intercorrelations amongst technology-related variables.

	No. of Devices Used	Post-Injury VC Use	No. of VC Platforms Used	Computer Anxiety (*n* = 180)	Computer Self- Efficacy (*n* = 184)	Technology Fluency (*n* = 184)
No. of Devices Used	*					
Post-injury VC Use	0.43 ****	*				
No. of VC Platforms Used	0.49 ****	0.72 ****	*			
Computer Anxiety	−0.52 ****	−0.38 ****	−0.45 ****	*		
Computer Self-Efficacy	0.44 ****	0.31 ****	0.39 ****	−0.66 ****	*	
Technology Fluency	0.57 ****	0.44 ****	0.55 ****	−0.79 ****	0.67 ****	*

Note. * *p* < 0.05, ****, *p* < 0.0001.

**Table 6:** Fit statistics for the Model of VC Readiness.

χ^2^	df	*p*	RMSEA (90% CI) (<0.08)	SRMR (<0.05)	NFI (≥0.95)	TLI (≥0.95)	CFI (≥0.95)
24.713	15	0.054	0.058 (0.025–0.126)	0.05171	0.954	0.965	0.981

## Data Availability

The raw data supporting the conclusions of this article will be made available by the authors on request.
